# Metagenome-assembled genomes of four novel bacterial species from Atlantic rainforest stream sediments in Brazil

**DOI:** 10.1128/mra.00336-26

**Published:** 2026-06-04

**Authors:** Leandro Pio de Sousa, Angie Alejandra Calderon Fajardo, Marcelo Mendes Brandão, Valeria Maia de Oliveira, Gustavo Quevedo Romero

**Affiliations:** 1 Laboratory of Multitrophic Interactions and Biodiversity, Department of Animal Biology, Institute of Biology, University of Campinas (UNICAMP) 28132, Campinas, São Paulo, Brazil; 2 Laboratory of Systemic and Integrative Biology, Center of Molecular Biology and Genetic Engineering, University of Campinas (UNICAMP) 28132, Campinas, São Paulo, Brazil; 3 Microbial Resources Division, Research Center for Chemistry, Biology and Agriculture (CPQBA), University of Campinas (UNICAMP) 28132, Campinas, São Paulo, Brazil; Montana State University, Bozeman, Montana, USA

**Keywords:** stream, sediments, MAGs

## Abstract

Here, we report draft genome sequences of four novel bacterial species from Atlantic rainforest stream sediments in southeastern Brazil. The genomes represent distinct lineages within Nitrospirota and Pseudomonadota (average nucleotide identity <95% to known species) and encode diverse metabolic capabilities, including nitrification, denitrification, and aromatic compound degradation.

## ANNOUNCEMENT

Tropical stream ecosystems are home to a vast array of microbial diversity, yet this diversity remains inadequately characterized at the genomic level (1). The Atlantic Rainforest biome in southeastern Brazil is notably underrepresented in sediment microbiome studies, despite its ecological importance and high levels of anthropogenic impact. Here, we present draft genome sequences of four novel bacterial species recovered from stream sediments collected along an environmental degradation gradient in São Paulo State, Brazil.

Sediment samples, five centimeters deep, were collected from preserved and degraded streams in the Baixada Santista (24° 11' 08" S 46° 47' 15" W) and Vale do Ribeira regions (24° 26' 34" S 47° 10' 50" W). Three replicate sediment cores per stream were pooled in 1 g of material, and DNA was extracted using the DNeasy PowerSoil Pro Kit (Qiagen). The DNA quality and quantity were determined by using a Qubit 2.0 fluorometer. A total of 50 ng DNA aliquots of each sample were sent to Novogene Corporation, Inc. (California, USA) for whole-genome shotgun metagenomic sequencing, which was done on the Illumina HiSeq platform with a paired-end format and a read length of 150 bp. A total of 180 million clean reads were generated. Quality-filtered reads were performed using the FastQC 0.12 program. Reads with N bases and quality threshold below 20 were removed. The assembly was made with MEGAHIT 1.0 (2), and metagenomic bins were recovered using MetaBAT2 v2.15 (3). Genome completeness and contamination were assessed with CheckM2 v1.0.1 (4), and taxonomic classification was performed using GTDB-Tk v2.3.0 (5) against the GTDB release 220 database (5). Average nucleotide identity (ANI) to the closest reference genomes was calculated using FastANI v1.33 (6). Genome annotation was conducted with Prokka v1.14.6 (7) and eggNOG-mapper v2.1.9 (8).

*Candidatus* designations and species/genus epithets were proposed by the authors following the conventions of the International Code of Nomenclature of Prokaryotes. Species names were coined to reflect geographic origin, ecological context, or distinctive metabolic characteristics of each lineage, and are independent of the identifier strings used for reference genomes in the GTDB database. All species mentioned here fall into the category of “putative new species.”

Four metagenome-assembled genomes representing novel species-level lineages were recovered. *Candidatus Nitrospira jureiensis* (429 contigs, 1.87 Mb, 67.4% complete, 1.4% contamination) belongs to the family Nitrospiraceae (see Fig. S1a at https://doi.org/10.6084/m9.figshare.32101195) and shows 85.7% ANI to *Nitrospira_F* sp015903945. The genome harbors putative ammonia monooxygenase (*amo*ABC), nitrite reductase (*nir*K), and complete urease (*ure*ABCEFG) genes, suggesting capacity for nitrification and nitrogen cycling. *Candidatus Pseudolabrys nicotinovorans* (524 contigs, 2.08 Mb, 50.6% complete, and 2.4% contamination) from the family Xanthobacteraceae (see Fig. S1b at https://doi.org/10.6084/m9.figshare.32101195) shows 78.6% ANI to the nearest reference and encodes extensive aromatic compound degradation pathways, including 26 dioxygenase genes and eight carbon monoxide dehydrogenase genes. *Candidatus Magnacarnifex odordissolutor* (828 contigs, 3.04 Mb, 57.6% complete, and 16.5% contamination), representing a putatively novel genus within Rhodobacteraceae (see Fig. S1c at https://doi.org/10.6084/m9.figshare.32101195), shows 85.3% ANI to known species and harbors complete denitrification genes (*nos*ZDFYL) and four trimethylamine methyltransferase genes, indicating potential for dissimilatory nitrate reduction and methylotrophic metabolism. *Candidatus Ergothioneinifer oxalotrophus* (623 contigs, 4.02 Mb, 81.3% complete, and 4.0% contamination), also representing a putatively novel genus within Burkholderiaceae (ANI 83.3%; see Fig. S1d at https://doi.org/10.6084/m9.figshare.32101195), harbors genes for ergothioneine biosynthesis and oxalate degradation.

These genomes offer new perspectives on the metabolic diversity of tropical stream sediment microbiomes and establish genomic references for underrepresented bacterial lineages from the Atlantic Rainforest biome. For a more detailed description, see Supplementary Material 1 and Table S1 at https://doi.org/10.6084/m9.figshare.32101195.

## Data Availability

Raw metagenomic reads have been deposited in the NCBI Sequence Read Archive under BioProject accession number PRJNA1429331 (SRR37485361, SRR37485358, SRR37485359, and SRR37485360). The Supplemental material has been deposited at https://doi.org/10.6084/m9.figshare.32101195.
